# Neuropsychological Assessment: Digital vs. Paper Comparability in Older Adults

**DOI:** 10.1093/geroni/igaf122.3440

**Published:** 2025-12-31

**Authors:** Claire Snyder, Cay Anderson-Hanley, Aydana Djoroeva, Connor Cavanaugh, Shaelyn Kelley, Catherine Dacey, Samantha Corman, Lily Schwarcz

**Affiliations:** Schenectady, New York, United States; Union College, Schenectady, New York, United States; Union College, Schenectady, New York, United States; Union College, Schenectady, New York, United States; Union College, Schenectady, New York, United States; Union College, Schenectady, New York, United States; Union College, Schenectady, New York, United States; Union College, Schenectady, New York, United States

## Abstract

The use of digital neuropsychological assessment has increased since the COVID-19 pandemic and continues to be essential (Crivelli, 2024). While digital administration may be more efficient, the accuracy of the results may be questionable (due to potential differences in performance, etc.). This study aimed to evaluate the comparability of brief neuropsychological tests in similar digital and paper forms. Participants completed two cognitive screening tests and two brief tests of executive function: the paper MoCA, and the digital Boston Cognitive Assessment (BoCA), as well as the paper Stroop task (“pStroop”), and an electronic version “eStroop,” respectively. A study conducted by Castelli et al. (2021) found that the paper cognitive screener: the Montreal Cognitive Assessment (MoCA), is a sensitive and specific test used to detect cognitive impairment. In this study, it was hypothesized that the performance of digital and paper-and-pencil assessments would be similar, evidenced by positive correlations. Twelve older adults with a mean age of 68.5 years (SD = 8.07) completed a combined lab protocol. Among older adults, the MoCA and the BoCA were significantly moderately correlated, r(10) = .62, p = .03, while the pStroop and eStroop were significantly strongly correlated, r(10) = .90, p = .002. This study aimed to determine if digital and paper-and-pencil neuropsychological tests performance would be comparable among brief screening tests. The results showed that for older adults, there was a moderate to strong correlation between paper-and-pencil and digital neuropsychological testing, suggesting that the use of electronic tests may, in some situations, be interchangeable.

